# Blocking Protein kinase C signaling pathway: mechanistic insights into the anti-leishmanial activity of prospective herbal drugs from *Withania somnifera*

**DOI:** 10.1186/1471-2164-13-S7-S20

**Published:** 2012-12-07

**Authors:** Abhinav Grover, Shashank Prakash Katiyar, Jeyaraman Jeyakanthan, Vikash Kumar Dubey, Durai Sundar

**Affiliations:** 1 Department of Biochemical Engineering and Biotechnology, Indian Institute of Technology (IIT) Delhi, Hauz Khas, New Delhi 110016, India; 2 Department of Bioinformatics, Alagappa University, Karaikudi, Tamil Nadu 630003, India; 3 Department of Biotechnology, Indian Institute of Technology Guwahati, Guwahati 781039, India

## Abstract

**Background:**

Leishmaniasis is caused by several species of leishmania protozoan and is one of the major vector-born diseases after malaria and sleeping sickness. Toxicity of available drugs and drug resistance development by protozoa in recent years has made Leishmaniasis cure difficult and challenging. This urges the need to discover new antileishmanial-drug targets and antileishmanial-drug development.

**Results:**

Tertiary structure of leishmanial protein kinase C was predicted and found stable with a RMSD of 5.8Å during MD simulations. Natural compound withaferin A inhibited the predicted protein at its active site with -28.47 kcal/mol binding free energy. Withanone was also found to inhibit LPKC with good binding affinity of -22.57 kcal/mol. Both withaferin A and withanone were found stable within the binding pocket of predicted protein when MD simulations of ligand-bound protein complexes were carried out to examine the consistency of interactions between the two.

**Conclusions:**

Leishmanial protein kinase C (LPKC) has been identified as a potential target to develop drugs against Leishmaniasis. We modelled and refined the tertiary structure of LPKC using computational methods such as homology modelling and molecular dynamics simulations. This structure of LPKC was used to reveal mode of inhibition of two previous experimentally reported natural compounds from *Withania somnifera *- withaferin A and withanone.

## Background

Leishmaniasis is an endemic disease prevalent in many parts of the world; mostly in countries like India, Bangladesh, Pakistan, Afghanistan, Nepal, East and North Africa, and Deserts in western Asia [1]. Leishmaniasis is responsible for the death of approximately 70,000 people each year worldwide [2]. It is caused by various species of intramacrophage protozoan *Leishmania *like *Leishmania donovani, Leishmania major, Leishmania mexicana *and *Leishmania panamensis *to name a few, and spread by the bite of sandfly [1]. Leishmaniasis is becoming the disease of attention and concern because in the last few decades *L.donovani *has developed drug-resistance and toxicity towards available drugs [3,4]. Hence, it has become inevitable to identify new drug targets and to develop novel drugs against *L.donovani *to cure Leishmaniasis.

Previous experimental study has shown that methanolic compounds from *Withania somnifera *(ashwagandha) possess *in vitro *anti-leishmanial activity [5,6]. Withaferin A has been identified as one of ashwagandha's prominent phytocompounds. It is a cell permeable steroidal lactone which has been shown to possess anti-leishmanial property [5] apart from many other pharmacological properties. Withaferin A belongs to a class of compounds from *Withania somnifera *collectively known as withanolides. These exhibit number of other therapeutic activities like anticancer [7-11], anti-herpetic [12] and neuronal regeneration property [13]. Unlike higher eukaryotes, withaferin A has been reported to induce apoptosis in leishmanial cells by targeting its protein kinase [6].

Protein kinases in mammalian cells are associated with many important cellular processes like gene activation, cell differentiation and release of neurotransmitters [6,14,15]. On one hand, the types and role of protein kinases are well studied in mammalian cells, while on the other hand, only scarce information is available about protein kinases of protozoans. Previous studies have proven that protozoan protein kinases differ from mammalian protein kinases both structurally and functionally [16]. These differences between mammalian and protozoan protein kinases render these kinases as potential drug targets [17]. For the purpose of ease, protein kinase in *Leishmania *has been termed as leishmanial protein kinase C (LPKC) [18]. Although previous studies have reported the inhibition of LPKC by methanolic compounds of ashwagandha plant, so far no study has been carried out which provides the mechanism of action and structural insights of the inhibition. Structure of LPKC has not yet been solved experimentally and unavailability of this structure of LPKC further limits the development of drugs against it. Structure-based drug designing is a popular approach to search inhibitors against a target protein but it requires information of three dimensional structure of the target [19,20]. In the absence of experimental tertiary structures of a protein, computational methods such as homology modeling and threading are capable of predicting protein structures [21]. In such scenario, computational methods can be used to predict the structure and active site of LPKC. Probing LPKC's mode of inhibition by pharmacologically active compounds of ashwagandha will broaden the prospects of drug development against leishmaniasis and this information can be used to screen large number of inhibitors against it more accurately and rapidly. Ashwagandha also contains another important compound known as withanone which is known to possess antitoxic activity against methoxyacetic acid in addition to its prominent anticancer properties [22,23]. Though withanone has not yet been tested against leishmaniasis experimentally, this study provides a computational proof of its possible inhibitory activity against LPKC.

## Computational methods

### Homology modeling

1262 amino acid-long protein sequence of LPKC (Accession no. CBZ31403) was retrieved from NCBI protein database in FASTA format. Position-Specific Iterated BLAST against PDB database was used to identify homologous protein structures of LODC [24-27]. There was complete absence of any homologous structure for the residual range ~0-660 and ~1030-1262 amino acids. LPKC sequence ranging from 650-1025 is a conserved protein sequence and contains the catalytic domain of serine/threonine protein kinase. Because of unavailability of homologous structure for initial and last region of LPKC and highly conserved nature of 650-842 amino acids, only conserved stretch was considered for modeling purpose using homology modeling approach. Crystal structures of human calcium/calmodulin-dependent protein kinase type-IV (2W4O) at 2.17 Å resolution and death-associated protein kinase-1 (2Y0A) at 2.6 Å resolution were selected as templates for homology modeling. 2W4O had an e-score of 3 × 10^-11 ^and 27% identity with protein sequence of query with an 87% coverage and 2Y0A showed 2 × 10^-5 ^e-core and 30% identity with 46% coverage of query. Homology model of LPKC using crystal structure of selected templates was built using multi-template protocol of MODELLER version 9.10 [28,29]. Discrete Optimized Protein Energy (DOPE ) [30] was applied to refine the loops of the generated models. Models were accessed on the basis of Modeler Objective Function, DOPE scores, verify3D score [31,32] and ERRAT score [33]. To select a model out of the several models generated by MODELLER, dope energy profile was generated for templates and models. Model possessing closest DOPE energy profile with template was selected for further studies. Selected model was further refined and stabilized using Molecular Dynamics (MD) simulations [34].

### Molecular dynamics simulations

Desmond Molecular Dynamics system [35,36] with Optimized Potentials for Liquid Simulations (OPLS) all-atom force field 2005 [37,38] was used to perform MD simulations of all proteins and ligand-bound complexes. Modeled protein structure and structures of protein-ligand complexes were first prepared using protein preparation wizard of Maestro interface [36]. Prepared structures were then uploaded in Desmond set up wizard for MD simulations. Preparation of protein structure includes the addition and optimization of hydrogens, generation of disulphide bonds, and removal of water molecules and capping of terminals. Prepared protein molecules were solvated with TIP4P water model in a cubic periodic boundary box to generate required systems for MD simulations. Systems were neutralized using appropriate number of counterions. The distance between box wall and protein complex was set to greater than 10Å to avoid direct interaction with its own periodic image. Energy of prepared systems for MD simulations was minimized up to maximum 5000 steps using steepest descent method until a gradient threshold ( 25 kcal/mol/Å) is reached, followed by L-BFGS (Low-memory Broyden-Fletcher-Goldfarb-Shanno quasi-Newtonian minimizer) until a convergence threshold of 1 kcal/mol/Å was met. The systems were equilibrated with the default parameters provided in Desmond. Further MD simulations were carried on the equilibrated systems for desired period of time at constant temperature of 300 K and constant pressure of 1 atm with a time step of 2fs. During the MD simulations smooth particle mesh Ewald method was used to calculate long range electrostatic interactions. Nine Å cut-off radius was used for coulombic short range interaction cutoff method. The modeled LPKC protein was prepared for MD simulations using the parameters described above. The system was then continuously simulated for a long time period of 15ns. Stability of docking of ligands into the modeled proteins were also investigated using MD simulations. All protein-ligand complexes were simulated for 10ns time period using similar parameters as described above.

The root mean square deviation (RMSD) for both the modeled protein and the docked ligands within the binding pocket of protein were calculated for the entire simulations trajectory with reference to their respective first frames. ROG and H-bond analyses were carried out for all the frames of 15ns MD simulation of LPKCL. The hydrophobic interactions and H-bonds were calculated using Ligplot program [39] where H-bonds were defined as acceptor-donor atom distances of less than 3.3 Å, hydrogen-acceptor atom distance of maximum 2.7 Å and acceptor-H-donor angle greater than 90°. During the MD simulations, H-bond fluctuations of ligand with protein were calculated using VMD software [40].

### Binding site identification

Binding site and catalytic site of LPKC were present in serine/threonine kinase domain, which is a conserved sequence. Same domain was also present in human calcium/calmodulin-dependent protein (2W4O) which has an experimentally solved tertiary structure along with an inhibitor. Template structure of 2W4O was superimposed over the modeled structure to know the location of conserved binding site of LPKC. To confirm the binding site of LPKCL, detected by superimposition, structure of LPKCL was submitted to CASTp server [41] and SiteMap module [42]. Both CASTp and SiteMap confirmed the accuracy of predicted binding site as the binding site revealed by superimposition located within the largest and highest ranked cavity.

### Virtual molecular docking of ligands with LPKC

Structure files of withaferin A [PubChem:265237] and withanone [PubChem: 21679027] were retrieved from the PubChem Compound database. Structure files of both the ligands were prepared using LigPrep's ligand preparation protocol [43]. LigPrep improved the dataset of small molecules by generation of tautomeric, stereochemical and ionization variations, as well as by performing energy minimization and flexible filtering. Similarly, modeled protein structures were also prepared before the docking steps using Schrödinger's protein preparation wizard [44]. Protein preparation implicated the addition and optimization of hydrogen atoms, removal of bad contacts, optimization of bond lengths, creation of disulphide bonds, capping of protein terminals, and conversion of selenomethionine to methionine. A grid was generated at the predicted binding site of modeled structure as an essential step for docking using the Glide docking module of Schrödinger [45,46].

Prepared natural compounds were virtually docked against modeled LPKC protein at desired grid coordinates using Glide model's XP docking protocols [45]. Stability of the top scoring docked conformations obtained from glide XP docking, was inspected using MD simulations. All the Glide docking studies were performed on Intel Core 2 Duo CPU @ 3 GHz of HP origin with 1 GB DDR RAM. Schrodinger 9 Maestro interface was compiled and run under Ubuntu 32 bits operating system. All the MD simulations studies were performed in GPU server Intel (R) Core (TM) i7 CPU 930, with 4 GB DDR RAM.

### Prime/MM-GBSA binding-free energy calculation

Binding free energies of complexes were calculated using Prime/MM-GBSA method [47-49]. Output post-viewer files of the XP docking protocol were used for the calculation of free energy of binding by Prime/MM-GBSA protocol. The binding free energy Δ*G*_binding _was estimated using following equation:

$$\Delta G_{\text{binding}}=E_{\text{R:L}}-\left(E_{\text{R}}+E_{\text{L}}\right)+\Delta G_{\text{soly}}+\Delta G_{\text{SA}}$$

where *E*_R:L _is the energy of the complex, *E*_R _+ *E*_L _is sum of energies of the receptor and ligand in unbound state, ΔG_solv _is the difference in the GBSA solvation energy of the complex and sum total of solvation energies of unbound receptor and ligand. ΔG_SA _is the difference in surface area energies of the complex and sum total of surface area energies of unbound receptor and inhibitor. OPLS-AA force field [38] and GB/SA continuum solvent model were used to calculate necessary energies of the complexes.

## Results and discussion

### LPKC protein structure modeling and active site prediction

Two homologous X-ray crystal structures of human calcium/calmodulin-dependant protein kinase and death associated protein kinase-1 were used as templates to predict the tertiary structure of LPKC protein. Predicted protein contained all the residues in allowed regions on Ramachandran plot and showed ~70% ERRAT score. Low resolutions of the template structures were the probable cause of slightly low ERRAT score of LPKC structure. Hence modeled protein was stabilized by MD simulations technique. LPKC is a kinase protein which possesses a conserved active site similar to other protein kinases. However, primary structure of LPKC is distinctly related to mammalian eukaryotic kinases, LPKC protein tertiary structure aligned well with human calcium/calmodulin-dependant protein kinase protein structure. Structural alignment of LPKC with 2W4O protein structure identified the plausible active site of LPKC protein which was further confirmed using cavity analysis server CASTp and active site identification software SiteMap. CASTp and Sitemap reported the presence of Asp666, Arg667, Gln669, Arg670, Glu687, Glu689, Gln691, Asn710, Val711, Thr712, Ala713, Leu714, Met728, Glu729, Ala731, Asp778, and Ser781 residues around the highest scoring cavity [Additional File 1].

### Molecular dynamics simulations of modeled LPKC protein

**Figure 1:**
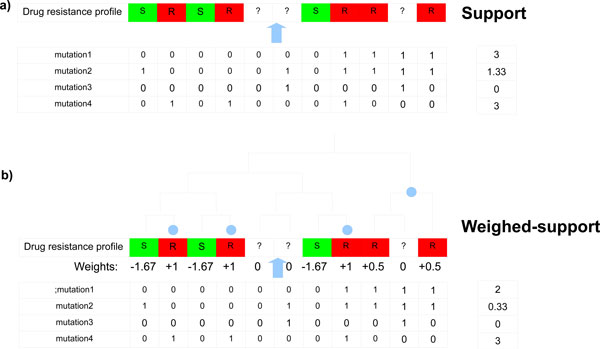
**RMSD trajectory of the modeled LPKC protein backbone during the 15ns long MD simulations**. Duration of MD simulations is scaled on X-axis and Y-axis represents the RMSD deviation of protein structure in Å.

To analyze the stability of predicted protein, RMSD of its backbone was plotted as the time dependant function of MD simulations [Figure 1]. Fluctuation in backbone of modeled protein during the simulations was recorded up to 5.87 Å. After 5ns of MD simulations, backbone was found to fluctuate around 5Å which persisted till the end of 15ns simulation. The standard deviation (SD) in RMSD for whole simulation process was found as 0.97 which was comparatively higher than 0.3 for the last 10 ns of simulation time. These data suggest that protein had more flexible backbone in the beginning of the MD simulations but as the simulations continued, protein tend to acquire a higher stable configuration. A low RMSD throughout the MD simulation and consistent RMSD at the last of MD simulation indicated that the predicted tertiary structure of LPKC had acquired a stable folding conformation. Measure of the radius of gyration (ROG) is considered as an indicator of compactness of the protein structure [50]. Hence, variation in ROG values of protein represented the variation in the compactness in the protein structure along the simulation.

**Figure 2:**
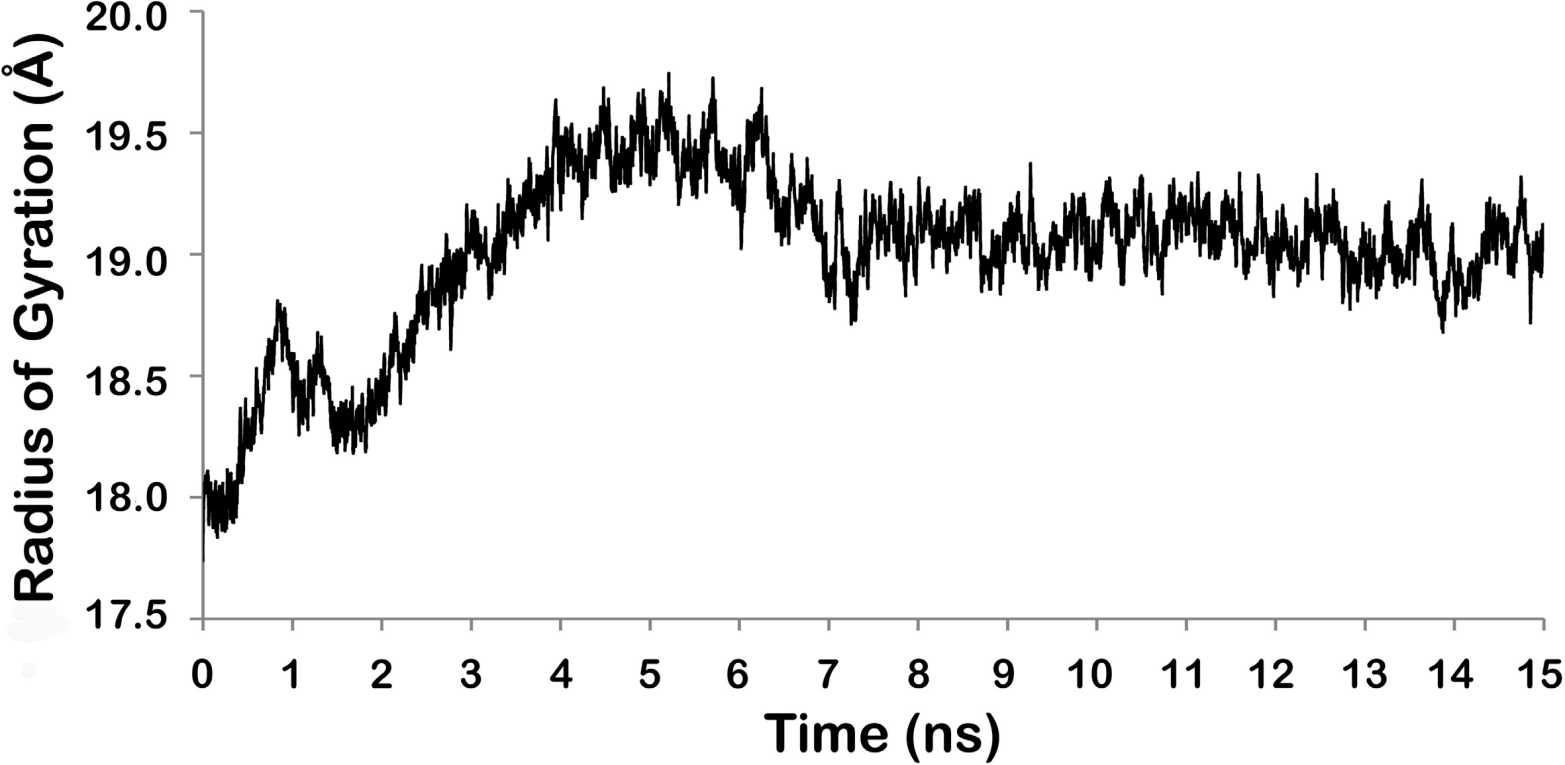
**RMSD in the ROG of LPKC protein during the 15ns long MD simulations**. Duration of MD simulations is scaled on X-axis and Y-axis represents the RMSD deviation of protein structure in Å.

**Figure 3:**
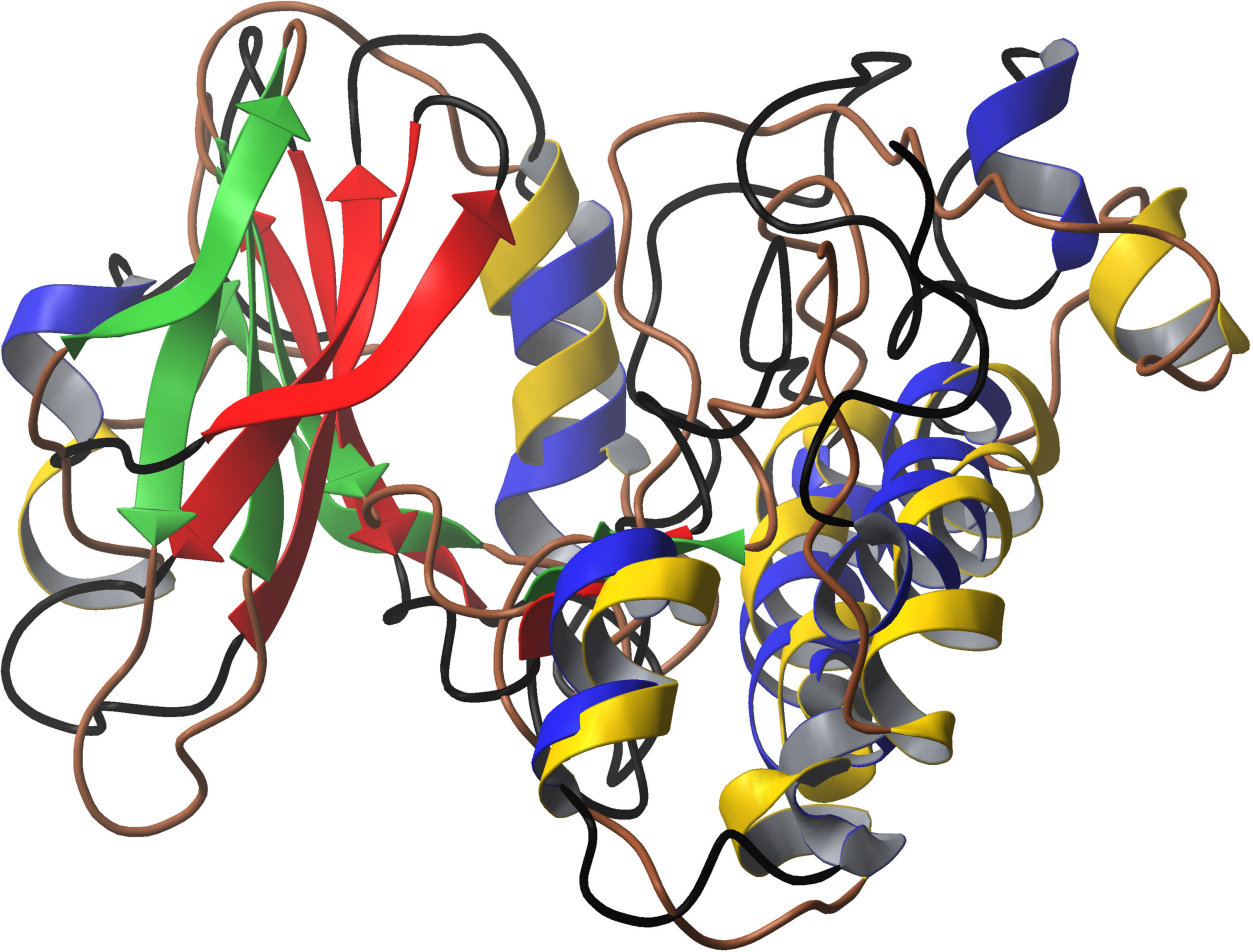
**All atom superimposition of modeled LPKC protein structure before and after the simulation**. Helices (blue and yellow), sheets (red and green) and loops (black and brown) are shown in different colors for first and last frame respectively.

**Figure 4:**
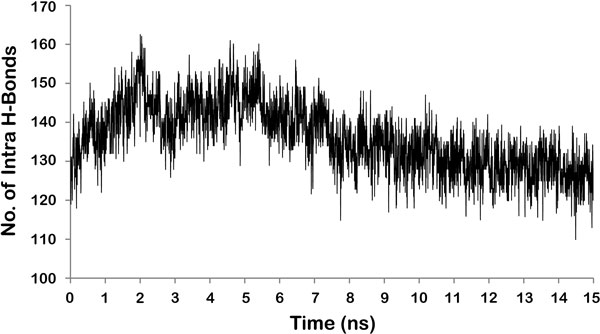
**Variation in number of Intra-H-bonds during the 15ns MD simulations of LPKC**.

Modeled protein had ROG value of around 18 Å before the simulation. ROG fluctuated slightly during initial 2 ns of simulations followed by higher fluctuations up to 7 ns [Figure 2]. After 7 ns, protein ROG settled around 19 Å, which is 1Å higher than the initial ROG value. Higher ROG of simulated structures than the initial modeled protein indicated that protein has expanded during the MD simulations in order to acquire more stable conformation. Increase in ROG can be clearly observed when initial and final structures are superimposed on each other [Figure 3]. Although ROG graph indicated that there has been an increase in the inter-atomic distances of protein during the simulations, consistent ROG of 19Å during the last 8 ns confirmed development of stable protein conformations. Intra-Hbonds of a protein during the MD simulations can provide important information about the stability of protein. Intra-Hbond profiling of LPKC protein [Figure 4] indicated that protein conformations during the last 8 ns had almost equal number of H-bonds as the starting conformation. It is clear from the Intra-Hbond graph [Figure 4] that all conformations of these 8 ns trajectory had equal number of H-bonds, which is consistent with respect to the RMSD graph of LPKC protein and supporting the fact that LPKC acquired a stable conformation during later part of the simulations. Considering all above observations in ROG, RMSD and intra H-bonds profiling collectively, it can be said that modeled structure was not one of the most stable confirmation of LPKCL, which is why it deviated from its native structure by ~5Å during the MDsimulation. However, after 7 to 8 ns of MD simulation, LPKCL reached to that conformational state which was more stable than the previous state hence after 7 or 8ns MD simulations, structure of LPKCL did not deviated further. The structural changes in LPKCL protein caused expansion of the protein structure, as suggested by the increased ROG of more stable conformations.

**Figure 5:**
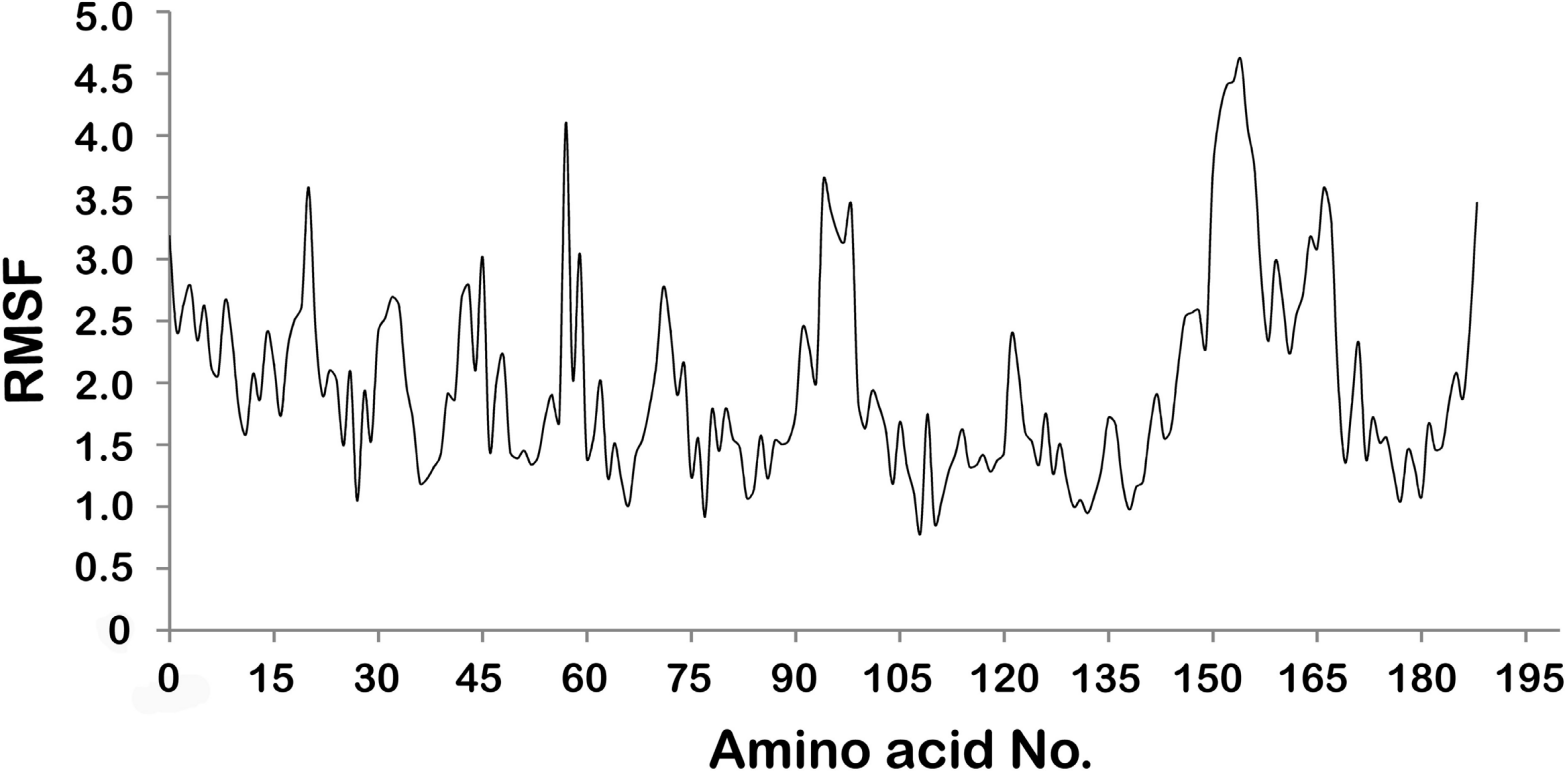
**Root Mean Square Fluctuation (RMSF) of all residues of LPKC protein during the MD simulations**.

Root Mean Square Fluctuation (RMSF) of all the residues was calculated during the 15ns MD simulations as well as for last 8ns to locate the regions of higher flexibility in the protein. In RMSF graph of LPKC for all frames [Figure 5], we can see that the region between residues 15-20, 90-100 and 150-160 have highest deviation during the MD simulations. Same pattern was observed in the RMSF graph during the last 8ns [Additional File 2] which was the stable phase of LPKCL MD simulations. These regions of higher variability belong to loop secondary structure of LPKC protein. It is a well known fact that loop region tends to be more flexible than other part of protein. During the 15 ns MD simulations of LPKC protein, a separate analysis to calculate the deviation in only the loop regions of protein revealed that these are the regions of higher flexibility of around 6.80 Å. Hence, it is clear from above analysis that high RMSF of few part of protein is caused by the loop structures.

### Virtual docking of protein with withaferin A and withanone

**Figure 6:**
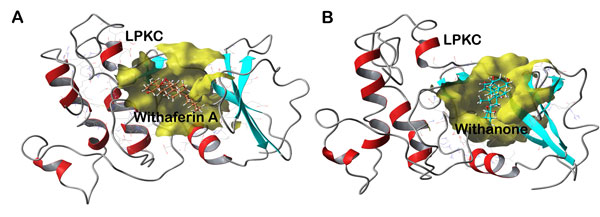
**Docking complexes of LPKC with natural compounds A) Withaferin A within the binding pocket of LPKC after virtual molecular docking**. B) Withanone within the binding pocket of LPKC after virtual molecular docking. Both ligands are shown to occupy the binding site.

Prepared structures of withaferin A and withanone were docked with stabilized structure of LPKC and both showed almost equal binding affinity with LPKC. Withaferin A and withanone bound to LPKC protein with a Gide XP score of -6.01 and -6.41 [Table 1]. Withaferin A has already been reported to bind with LPKC protein by experimental studies [6]. Figure 6 shows both these ligands being bound at their respective binding pockets during the docking. As scoring functions of docking programs are not exclusively reliable [51], validation of the docking results are needed to be performed by other reliable approaches such as free energy calculations and MD simulations of docked complexes. PRIME Free energy of binding for withaferin A-LPKC complex was measured as -28.47 kcal/mol while -22.57 kcal/mol for withanone-LPKC complex. Contrary to the docking scores, withaferin A was found to show higher binding affinity with LPKC as compared to withanone. There was less difference between the values of docking score and between values of free energy, indicating that both withaferin A as well as withanone bind to LPKC with almost equal affinity.

**Table 1:** XP Docking scores and Binding energies of LPKC with natural compounds

Complex	XP Glide Score	Prime/MM-GBSA binding-free energy (dG)(kcal/mol) (BeforeMDS/After MDS)
Withaferin A-LPKC complex	-6.01	-28.47/-17.97
Withanone-LPKC complex	-6.41	-22.57/-18.31

### Molecular dynamics simulations of complexes

Stability of both the natural compounds in the binding pockets of LPKC was further analyzed by MD simulation. RMSD analysis of withaferin A during 10 ns simulations showed that withaferin A altered its configuration by 1Å at very beginning of the simulation and maximum RMSD of 1.36 Å was noticed at 7.7 ns and that too for just one frame. After the initial deviation, withaferin A did not deviate further and showed consistent RMSD of around 1Å throughout the simulation process indicating that withaferin A had acquired a very stable conformational state.

**Figure 7:**
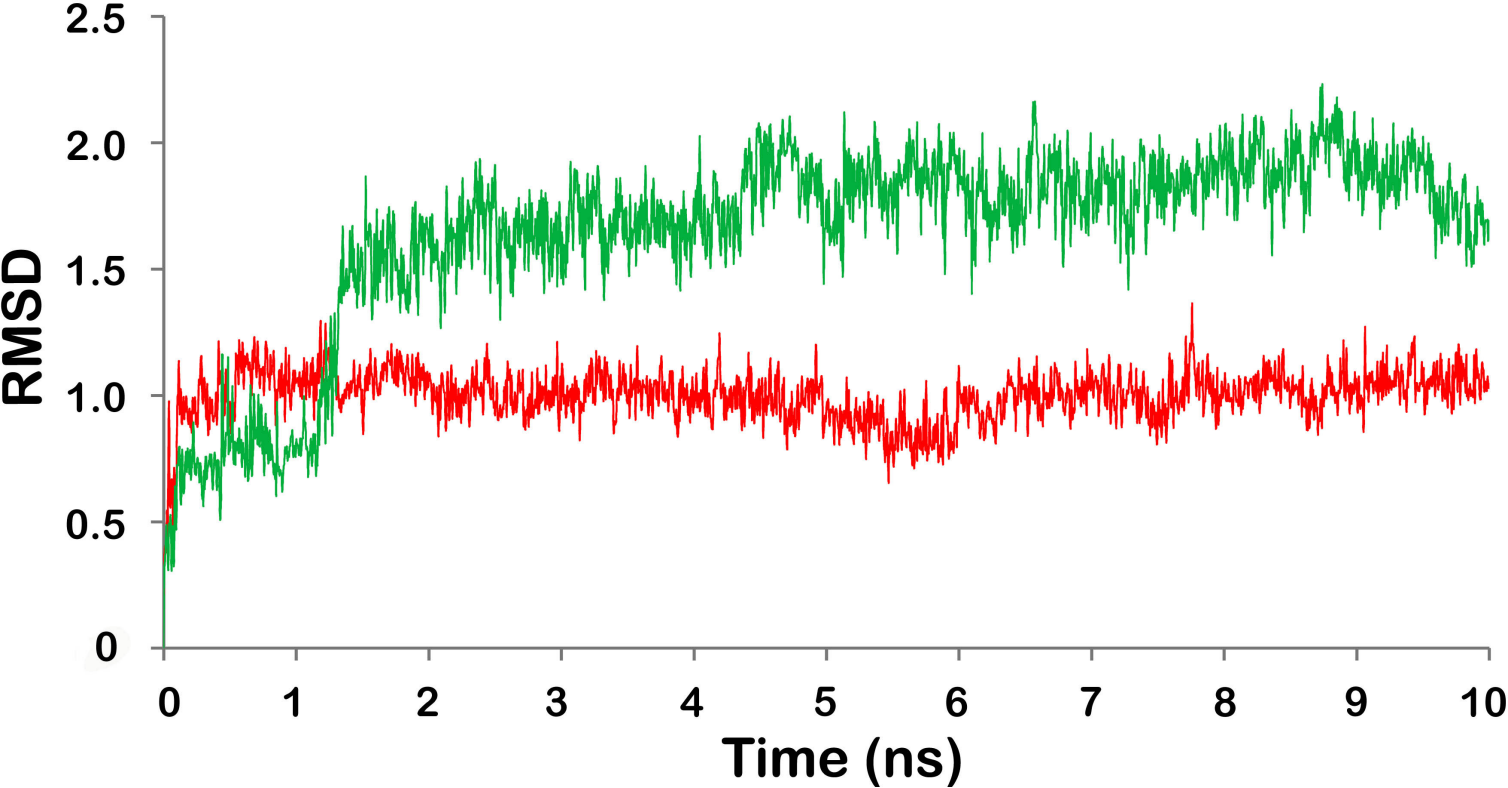
**RMSD trajectory of the withaferin A in withaferin A-LPKC complex (red) and withanone in withanone-LPKC complex (green)**. Duration of MD simulations is scaled on X-axis and Y-axis represents the RMSD deviation of protein structure in Å.

Withanone showed higher RMSD as compared to withaferin A during the 10 ns MD simulations [Figure 7]. Withanone deviated by 1.5 Å after ~1.5 ns MD simulations with a maximum RMSD of 2.2 Å. After 1.5 ns simulation, Withanone showed consistent RMSD of around 1.5 Å throughout the MD simulations which points towards its stability during the simulations.

### Interaction analysis of withaferin A and withanone with LPKC protein

**Figure 8:**
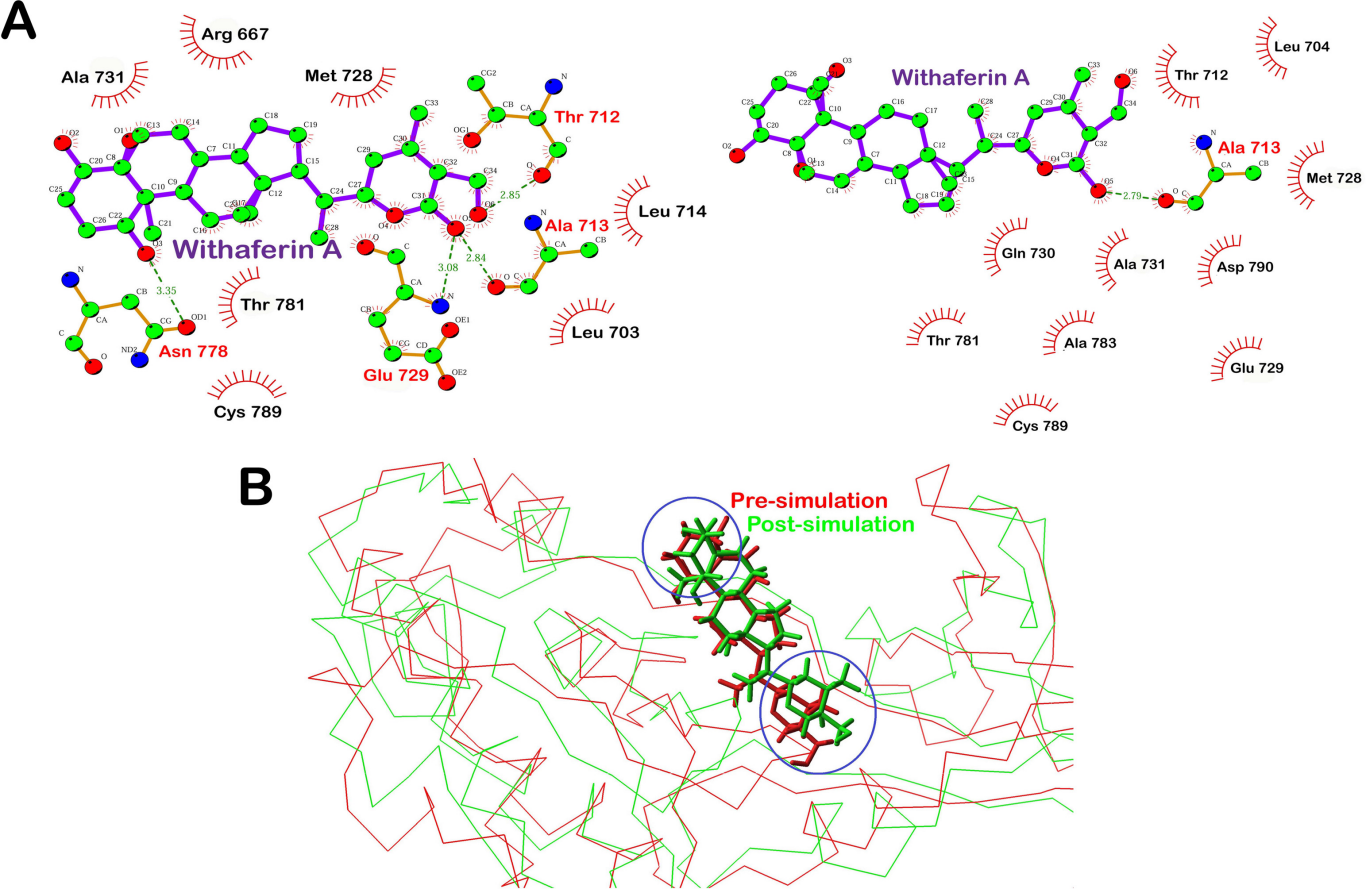
**Interactions between LPKC and withaferin A drawn by Ligplot**. Figure shows the changes in interactions and conformation of bound withaferin A with LPKC during 10ns MD simulations. **A) **Change in interaction profile of withaferin A-LPKC complex during the MD simulations. **B) **Superimposed withaferin A-LPKC complex structures before the simulation (red) and after the simulation (green). Blue circles specify the regions of conformational change in withaferin A structure.

After docking, both withaferin A and withanone were able to bind with the residues around active site of LPKC. The interaction profile of both natural compounds with LPKC protein residues has been described in Table 2. Withaferin A interacted with Thr712, Ala713, Glu729 and Asn778 via H-bonds and formed hydrophobic interactions with Arg667, Leu703, Leu714, Met728, Ala731, Thr781 and Cys789 residues of LPKC [Figure 8A and Additional File 3]. After the simulations of withaferin A-LPKC complex, withaferin A interacted with Ala713 via H-bonds and with Leu704, Thr712, Met728, Glu729, Ala731, Gln735, Thr781, Ala783, Cys789 and Asp790 by hydrophobic intractions [Figure 8A]. H-bond between O5 of withaferin A and O of Ala713 was conserved during the simulation with bondlength between 2.79 Å to 2.84 Å, indicating the stability of this bond. Stable interactions such as H-bonds and hydrobhobic interactions between protein and ligand strengthen the binding affinity. Similar to H-bonds, many hydrophobic interaction were also persistent during the simulation such as those with Thr712, Met728, Gly729, Ala731 and Thr781. Most of the residues involved in hydrophobic interactions and prefer to form van der Waals contacts. These are the residues around the active site of LPKC and occupation of binding sites of these residues by ligand instead of its natural substrate will lead to inhibition of protein function. There was loss of few hydrogen bond interactions between withaferin A and LPKC protein during the simulations but all those residues which were interacting via H-bonds previously, were found to form hydrophobic interactions after the MD simulations. Free energy of binding calculated for the last frame of MD simulations of withaferin A-LPKC complex, was -17.97 Kcal/mol which was lower than the pre-MD simulations complex. These results indicated that there has been changes in binding affinity of withaferin A during the MD simulation. It is clear from RMSD graph of withaferin A [Figure 7] that there has been a slight change in the conformation of withaferin A during the MD simulations in order to stabilize the ligand within the binding pocket of LPKC. Though the process of stabilization led to the disappearane of few hydrogen bonds, there has been substantial increase in the hydrophobic interactions. Superimposition of initial frame over last frame of withaferin A-LPKC complex revealed that conformational change in withaferin A structure has been brought by certain part of it which was involved in H-bond interaction [Figure 8B]. Probably, change in its conformation within the binding pocket of LPKC was caused by loss of H-bonds between certain parts of withaferin A and protein.

**Table 2:** Interaction profile of LPKC with natural compounds

Type of Interaction	Withaferin A (before MD)	Withaferin A (after MD)	Withanone (before MD)	Withanone (after MD)
**H-bonds**	Thr62, Ala63, Glu79, Asn128	Ala63	Ala63, Glu79	Glu79
**Hydrophobic**	Arg17, Leu53, Leu64, Met78, Ala81, Thr131, Cys139	Leu54, Thr62, Met78, Glu79, Ala81, Gln85, Thr131, Ala133, Cys139, Asp140	Arg17, Gln19, Glu39, Leu53, Thr62, Leu64, Met78, Ala81,	Gln19, Thr62, Met78, Ala81

**Figure 9:**
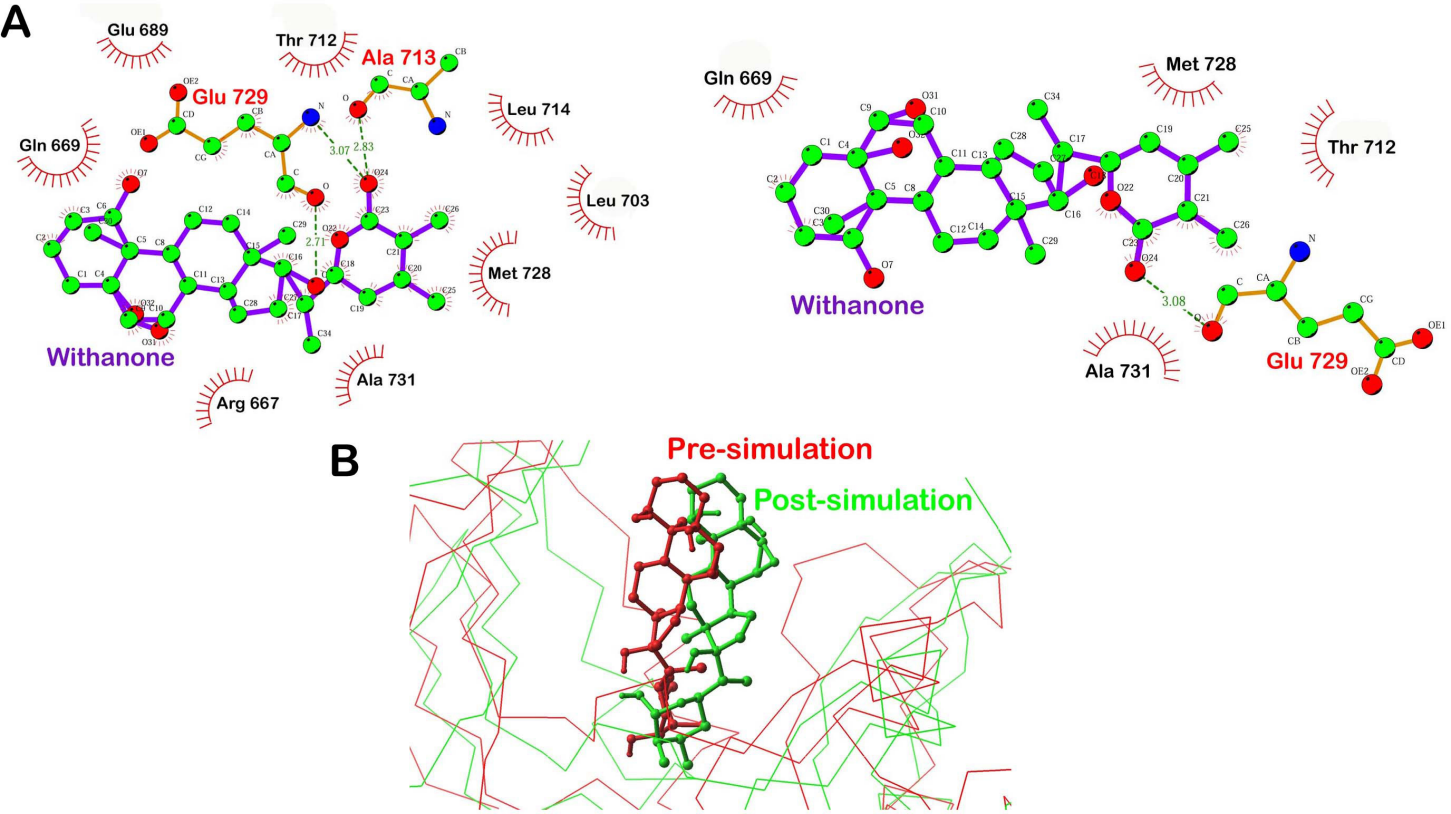
**Interactions between LPKC and withanone drawn by Ligplot**. Figure shows the changes in interactions and conformation of bound withanone with LPKC during 10ns MD simulations. **A) **Change in interaction profile of withanone-LPKC complex during the MD simulations. **B) **Superimposed withanone-LPKC complex structures before the simulations (red) and after the simulations (green).

Withanone was forming H-Bonds with Ala713 and Glu729 after the XP docking and before MD simulations. After the simulations, H-bond with Ala713 disappeared while Glu73 was still in contact with withanone via H-bond [Figure 9A]. Residues Arg667, Gln669, Glu689, Leu703, Thr712, Leu714, Met728 and Ala731 of LPKC formed hydrophobic interactions with withanone after the docking but only residues Gln669, Thr712, Met728, and Ala731 were found in contact with withanone post MD simulations [Figure 9A and Supplementary Additional File 4]. RMSD analysis of withanone during withanone-LPKC MD simulations indicated change in withanone's conformation by 1.5Å which persisted till the end of the simulations, as can be clearly seen by the superimposition of pre-MD simulations conformation of withanone-LPKC complex over post-MD simulated conformation [Figure 9B]. Withanone was found more flexible than withaferin A during the MD simulations which can be seen during the RMSD analysis of withanone and comparison of interaction profile before the simulation and after the simulation by LigPlot analysis. One H-bond disappeared during the simulaitons of withanone- LPKC complex. However, H-bond between O atom of Glu739 and O24 of withanone was conserved during the simulation with same bond length of ~3.07 Å. Many hydrophobic interactions between withanone and LPKC active site residues were also found persistent during the simulations such as those with Glu669, Thr712, Met728 and Ala731. Perseverance of interaction between protein and ligand during the simulations kept withanone intact within the binding pocket of LPKC protein. Post MD simulations, free energy of withanone-LPKC complex was found to be -18.31 Kcal/mol indicating a slight fall in the binding affinity [Table 1]. This data suggests that during the MD simulations, withanone lost few interactions with LPKC which led to change in its conformation and caused lowering of binding affinity but finally resulted in acquiring a stable conformation.

Though both withaferin A and withanone were found stable within the binding pocket of LPKC during the MD simulations, higher RMSD was observed for withanone as compared to withaferin A during the MD simulation but both had acquired a conformation which was not deviating any longer at the end of MD simulation. There was not a noticeable difference between the docking score of Withaferin A and Withanone after the docking and after the MD simulations. Both withaferin A and withanone were also found to interact with same residues of the active site after the docking process either via H-bonds or hydrophobic interactions, thus validating the accuracy of predicted binding pocket of LPKC and also confirming the inhibitory nature of both natural compounds against the kinase. Comparison of final binding free energies of LPKC complexes with withaferin A and withanone suggested that both show almost similar free energies of binding [Table 2].

## Conclusions

Withaferin A and withnone are two pharmacologically active natural products from the medicinal plant *Withania somnifera*. Withaferin A has been reported to exhibit antileishmanial properties in previous studies. We analyzed the inhibitory property of withaferin A as well as that of withanone at the molecular level. We modeled an important enzyme of leishmania - LPKC using comparative homology modeling and virtually docked withaferin A and withanone with it. Both withaferin A and withanone were found to inhibit LPKC protein with almost equal affinity. Withanone has not yet been experimently proven to inhibit LPKC protein before. The present study suggests that these two natural products can be potential candidates for checking Leishmaniasis by inhibiting LPKC. By this study we provide structural insights of the inhibitory action of withaferin A and withanone against LPKC.

## Competing interests

The authors declare that they have no competing interests.

## Supplementary Material

Additional File 1 **Active site of LPKCL identified by superimposed ligand and SiteMap analysis**. Superimposed ligand (DKI 1338) over LPKCL from protein 2W4O is shown in CPK molecular representation. Mesh like structure is the top predicted site by SiteMap software. It is clear from the picture that superimposed ligand lies perfectly within the predicted binding pocket. Same binding pocket was used for the grid generation. (*.jpg). Click here for file

Additional File 2 **RMSF graph generated only for last 8ns MD simulation**. (*.jpg). Click here for file

Additional File 3 **WithaferinA is bound at the binding pocket of LPKCL**. A) Withaferin-A surrounded by active site residues within its correct binding site. B) A ligand-receptor interaction diagram is shown to look at interacting residues within the radius of 4 Å. (*.jpg). Click here for file

Additional File 4 **A view of bound Withanone at the binding pocket of LPKCL**. A) Withanone surrounded by active site residues within its correct binding site. B) A 2D ligand-receptor diagram is shown to look at interacting residues within the radius of 4 Å. (*.jpg). Click here for file
