# Supplementary figures and images for: Blocking Protein kinase C signaling pathway: mechanistic insights into the anti-leishmanial activity of prospective herbal drugs from Withania somnifera
Source: BMC Genomics. 2012 Dec 7;13(Suppl 7):S20. doi: 10.1186/1471-2164-13-S7-S20 (PMC3521472; doi:10.1186/1471-2164-13-S7-S20)

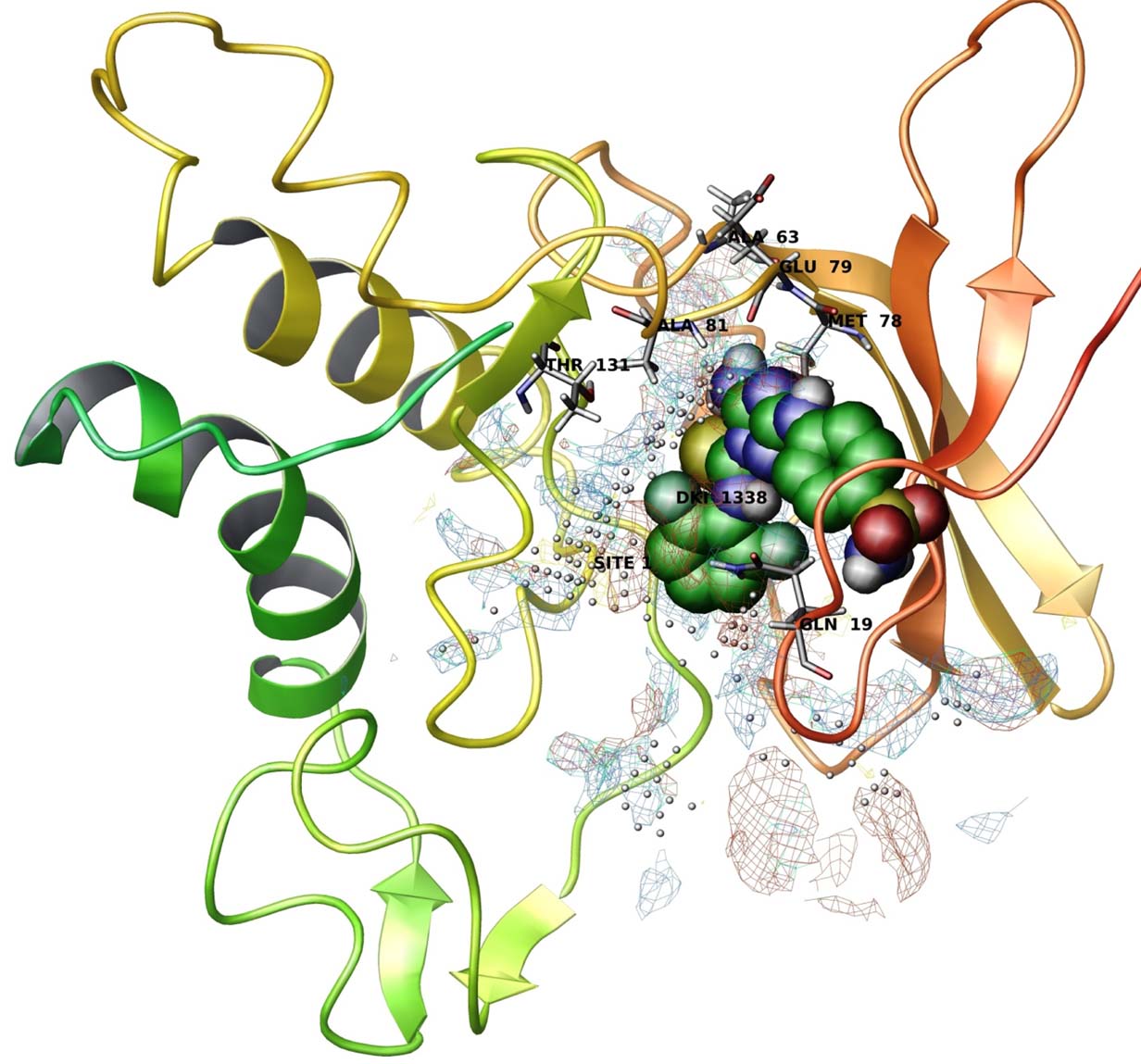

Supplement: Additional File 1 — Active site of LPKCL identified by superimposed ligand and SiteMap analysis. Superimposed ligand (DKI 1338) over LPKCL from protein 2W4O is shown in CPK molecular representation. Mesh like structure is the top predicted site by SiteMap software. It is clear from the picture that superimposed ligand lies perfectly within the predicted binding pocket. Same binding pocket was used for the grid generation. (*.jpg). [file 1471-2164-13-S7-S20-S1.jpg]

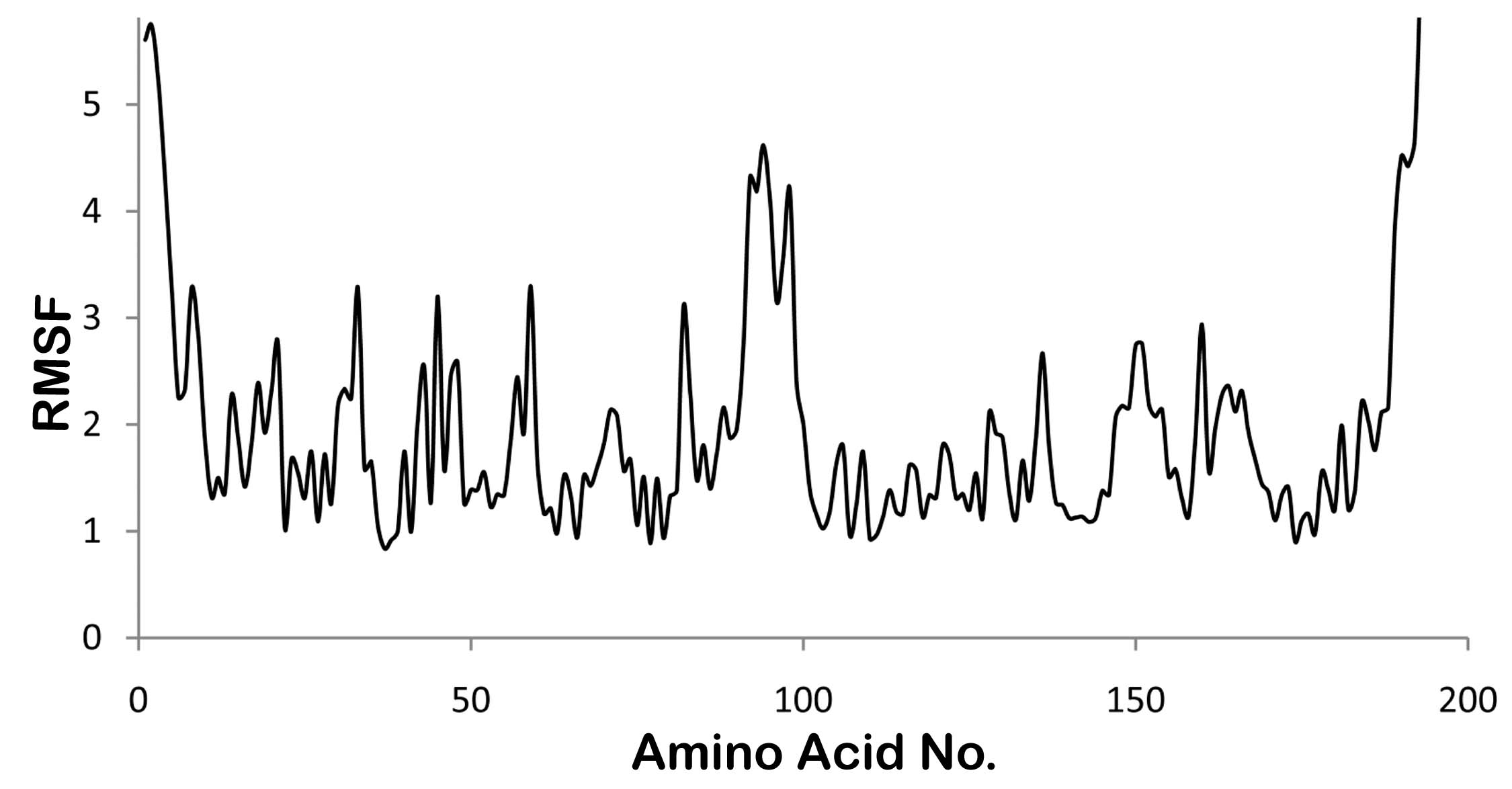

Supplement: Additional File 2 — RMSF graph generated only for last 8ns MD simulation. (*.jpg). [file 1471-2164-13-S7-S20-S2.jpg]

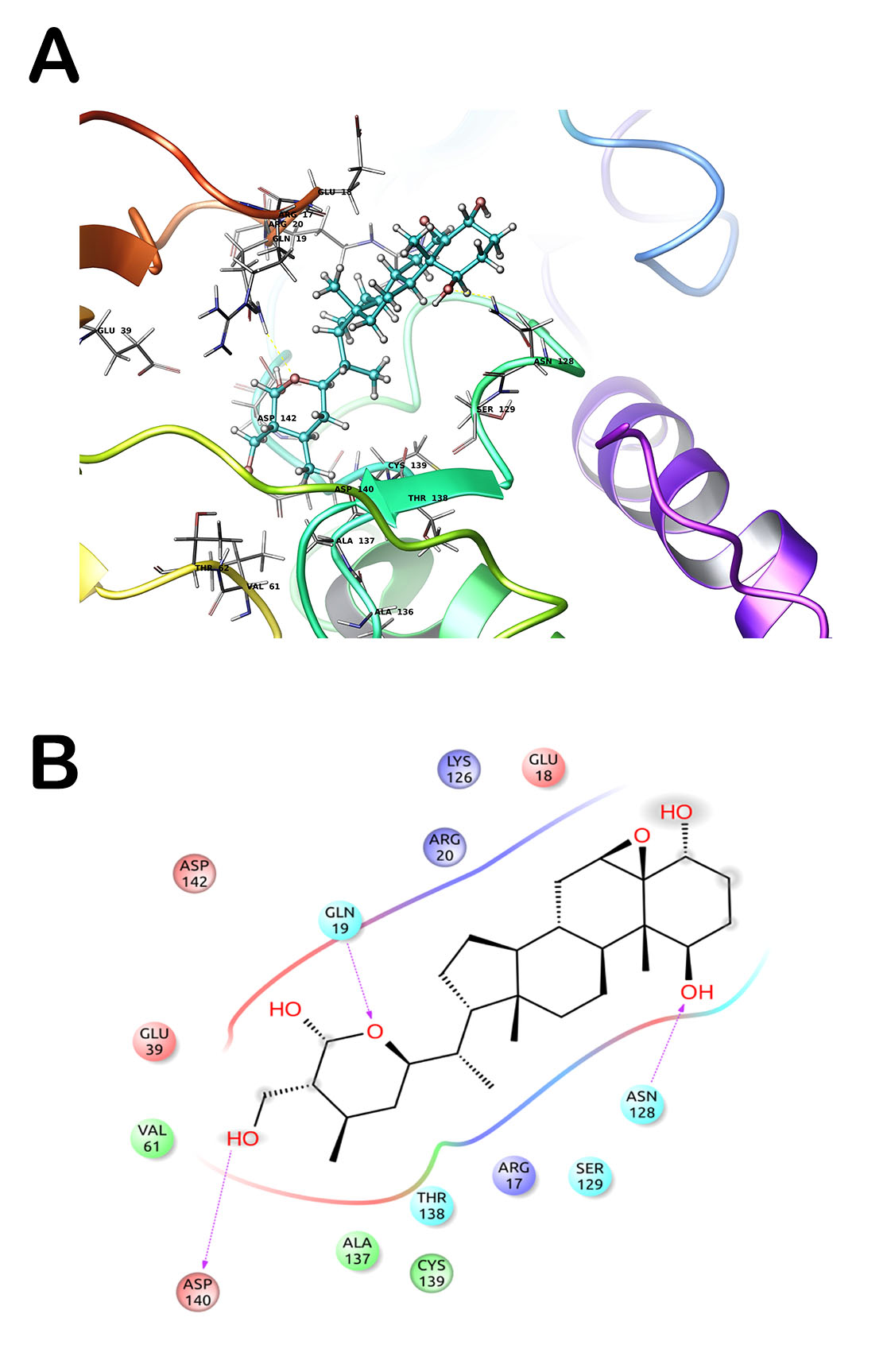

Supplement: Additional File 3 — WithaferinA is bound at the binding pocket of LPKCL. A) Withaferin-A surrounded by active site residues within its correct binding site. B) A ligand-receptor interaction diagram is shown to look at interacting residues within the radius of 4 Å. (*.jpg). [file 1471-2164-13-S7-S20-S3.jpg]

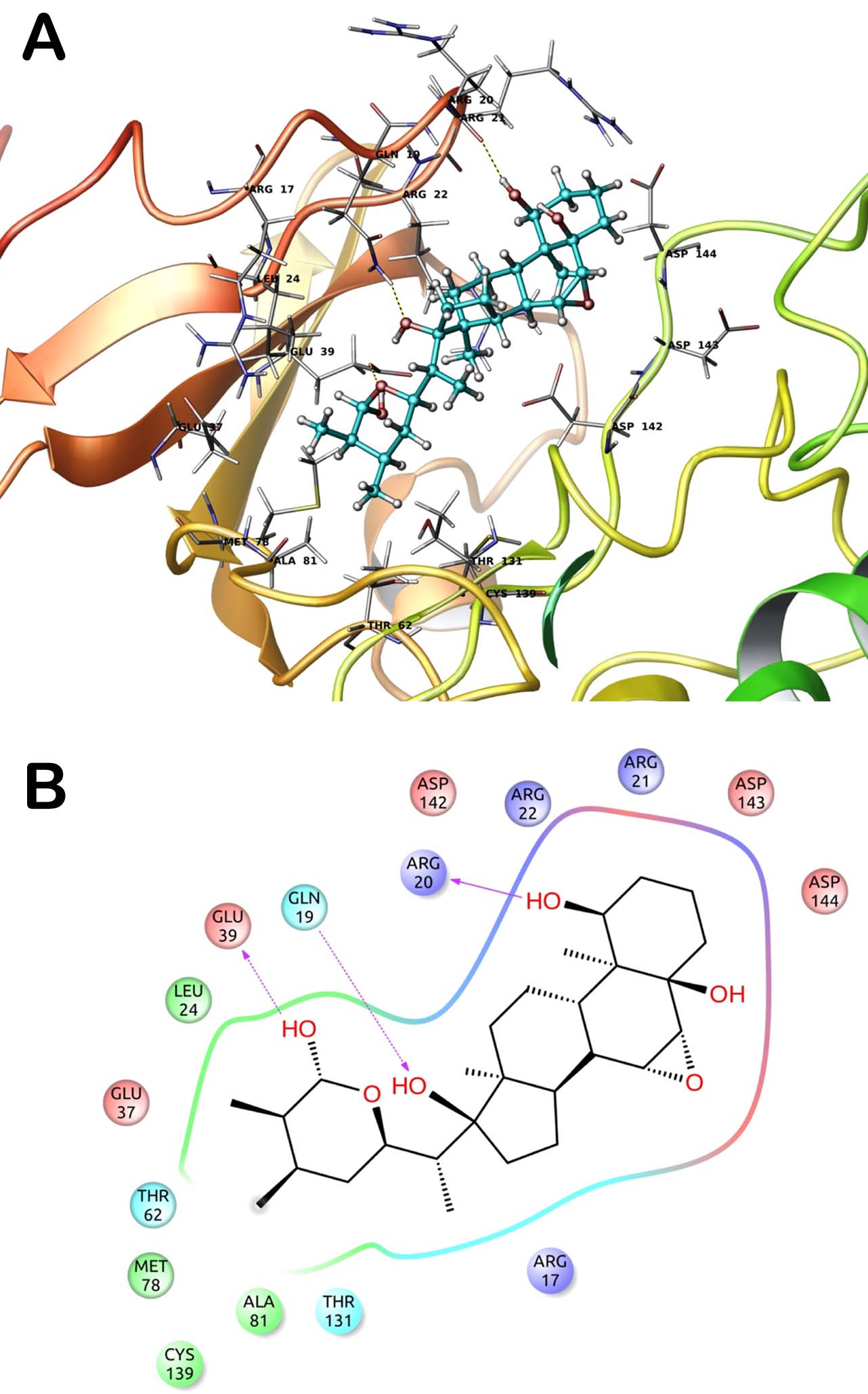

Supplement: Additional File 4 — A view of bound Withanone at the binding pocket of LPKCL. A) Withanone surrounded by active site residues within its correct binding site. B) A 2D ligand-receptor diagram is shown to look at interacting residues within the radius of 4 Å. (*.jpg). [file 1471-2164-13-S7-S20-S4.jpg]
